# A Do-It-Yourself Hyperspectral Imager Brought to Practice with Open-Source Python

**DOI:** 10.3390/s21041072

**Published:** 2021-02-04

**Authors:** Kimmo Aukusti Riihiaho, Matti Aleksanteri Eskelinen, Ilkka Pölönen

**Affiliations:** Faculty of Information Technology, University of Jyväskylä, P.O. Box 35, FI-40014 Jyväskylä, Finland; matti.a.eskelinen@gmail.com (M.A.E.); ilkka.polonen@jyu.fi (I.P.)

**Keywords:** spectral smile, optical aberration, aberration correction, do it yourself, open-source, hyperspectral imager

## Abstract

Commercial hyperspectral imagers (HSIs) are expensive and thus unobtainable for large audiences or research groups with low funding. In this study, we used an existing do-it-yourself push-broom HSI design for which we provide software to correct for spectral smile aberration without using an optical laboratory. The software also corrects an aberration which we call tilt. The tilt is specific for the particular imager design used, but correcting it may be beneficial for other similar devices. The tilt and spectral smile were reduced to zero in terms of used metrics. The software artifact is available as an open-source Github repository. We also present improved casing for the imager design, and, for those readers interested in building their own HSI, we provide print-ready and modifiable versions of the 3D-models required in manufacturing the imager. To our best knowledge, solving the spectral smile correction problem without an optical laboratory has not been previously reported. This study re-solved the problem with simpler and cheaper tools than those commonly utilized. We hope that this study will promote easier access to hyperspectral imaging for all audiences regardless of their financial status and availability of an optical laboratory.

## 1. Introduction

Hyperspectral imaging based on unmanned aerial vehicles (UAVs), especially drones, has gain a lot of attention in recent years, for example, in precision agriculture [1,2] and precision forestry [3,4,5,6]. Compared to more traditional aircraft- and spacecraft-based operations, drones offer ease of operation, cost-efficiency, and small ground-pixel size. In addition, drone-based imaging is possible even in cloudy weather [2].

Commercial hyperspectral imagers (HSIs) are expensive, which restricts their usage for low-budget operators, such as individual researchers or landowners, as even the cheapest HSIs cost tens of thousands of euros [7,8]. Commercial HSIs are also heavy and bulky, which introduces additional requirements for drones utilized in UAV campaigns [8]. Several cheap HSIs have been introduced in the literature: some are made lightweight for UAV operations [8,9], while others target laboratory usage [7]. While several of these HSIs can be manufactured in do-it-yourself manner, calibration and aberration correction methods usually require access to an optical laboratory.

**Figure 1:**
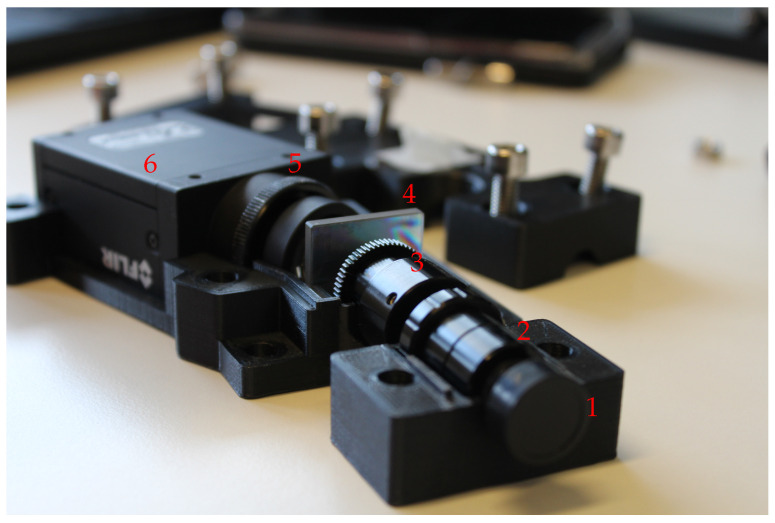
Our version of the Sigernes design push-broom imager with the top half removed. Light enters through the front lens (1). Connected to it is the series of S-mounts that contains the slit and the field lens (2), and the S-mount focus tube and the collimator lens (3). Diffraction grating (4) splits the light into its wavelengths. The detector lens (5) is connected to the machine vision camera (6).

Our study builds on do-it-yourself HSI presented by Sigernes et al. [9], which we call the Sigernes design. It is a push-broom type HSI that can be manufactured with off-the-shelf optical components and cameras, which are embedded in a 3D-printed casing. Its manufacturing process does not require access to special equipment other than a thermoplastic 3D printer. A photograph of our adaptation of the Sigernes design is shown in Figure 1.

A push-broom HSI forms a hyperspectral image by scanning the area being imaged line by line. The front lens sees a circular area, which is restricted to a line by a narrow slit. Light passing through the slit is collimated before diffracting it into its wavelengths by a diffraction grating. Finally, diffracted light is focused to the sensor of a video camera. In each captured frame, each row represents a spectrograph of a slit pixel. Stacking all scanned frames together forms the final hyperspectral image, often called an image cube. The image cube then consists of two spatial dimensions and one spectral dimension.

Manual manufacturing process of the Sigernes design gives rise to a certain aberration that we call the tilt. The tilt originates from misalignment of the slit, i.e., as the slit is cut into a circular plate of metal that is inserted by hand, it is very difficult to align it perfectly with the grating and the camera. In our experience, 1–2 degree accuracy can be achieved with reasonable effort. As the tilt is specific for this design, we did not find any suggestions how to correct it in the literature, although Sigernes et al. [9] noted its existence.

Aside from the problems caused by the manufacturing process, there exist several optical aberrations that are unavoidable in push-broom imagers in general. On an ideal push-broom HSI, given an evenly illuminated area of single material, any spectral emission lines would form perfectly vertical lines of even length on the sensor. On real devices, the lines are not straight or of even length, if not corrected by optical design or software solutions [10].

**Figure 2:**
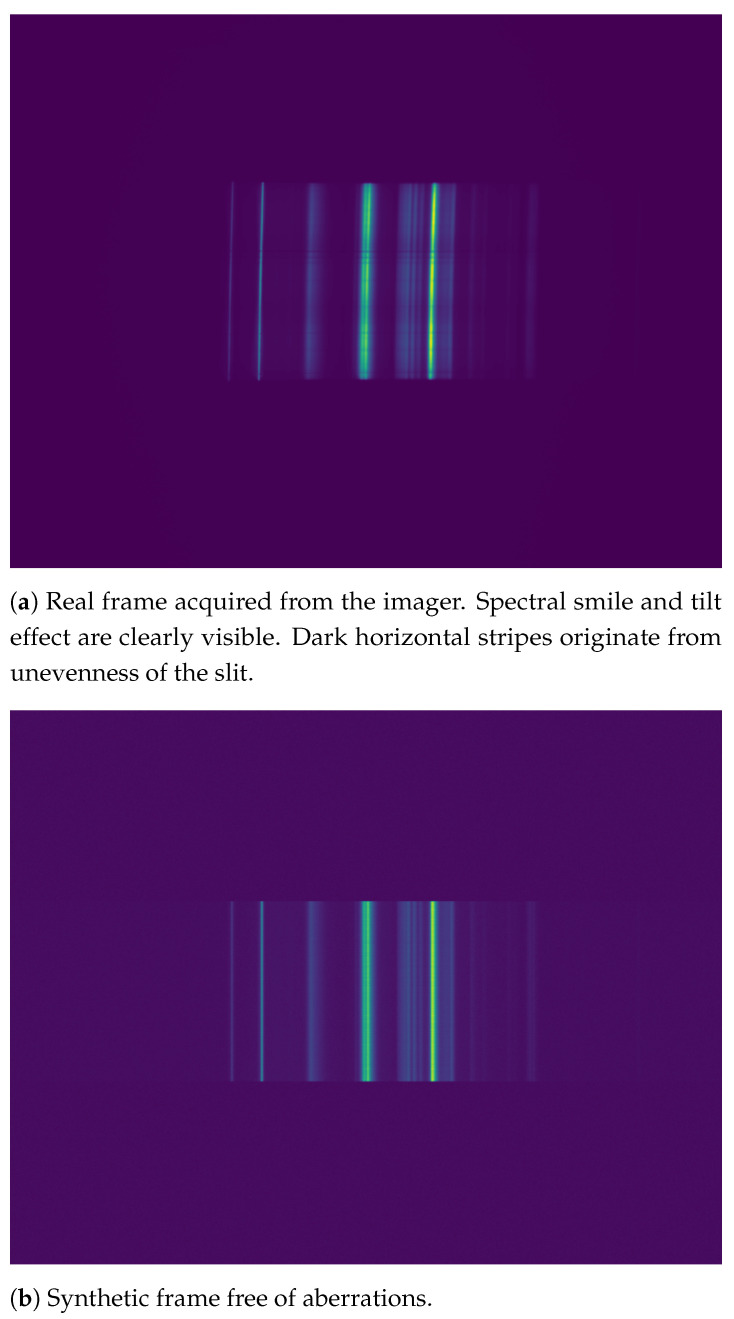
Real frame with aberrations and ideal synthetic frame compared side by side. The used imaging target was a fluorescence lamp.

Spectral smile is an aberration that causes the emission lines to have a curved shape. It is caused by dispersion when the slit image is spread over the sensor [11]. Figure 2 shows an example of a raw, uncorrected frame acquired from our imager side by side with an ideal synthetic frame that is free of aberrations. In addition, the well-known keystone aberration is barely visible in the real frame. It causes the emission lines to shorten towards the red end (right-hand side in the figure) of the spectrum. Tilt and smile aberrations are more easily distinguishable in figures in Section 3.

Optical aberrations are usually expressed in terms of point spread functions (PSFs) [10,12], but determining a PSF for a given instrument is not a trivial task and requires laboratory equipment, such as a monochromator [12] or spectral calibration lamps [13]. There exist several corrections for spectral smile [11,12,13,14], but we found none that could be carried out without an optical laboratory. For illustration of the most common optical aberrations present in push-broom imagers and suggestions on how to minimize their effect by optical design, please refer to [10]. For correction of keystone and smile of the Sigernes design when optical laboratory is available, please refer to [13].

In Section 2, we provide an improved casing for the Sigernes design as a printable 3D model (available at the project’s OSF repository at https://osf.io/3uhkb) along with an open-source software artifact capable of imaging and aberration correction (available at https://github.com/silmae/desmiler). Section 3 presents two experiments: the first one shows the effectiveness of our correction method with synthetic data, and the second one demonstrates imaging without an optical laboratory (dataset for experiment replication is also available at the OSF repository). The results of the experiments are shown in Section 4 and discussed in Section 5. Section 6 concludes the study.

## 2. Materials and Methods

The working principle of the Sigernes design is quite simple: light is first gathered and restricted to a narrow beam, which is then diffracted over the video camera’s sensor. The 3D-printed casing keeps the optical components immobile relative to each other and prevents stray light entering the sensor. The design of the optics is thoroughly described in [9]. Here, we highlight some changes and improvements made to the original casing. The presented 3D models are available at https://osf.io/3uhkb. Modifiable versions of the models can be opened and modified using the open-source computer aided design (CAD) software FreeCAD (https://www.freecadweb.org).

The slit needs to be aligned with the grating and the camera sensor in order to obtain correct spectral data. Any misalignment causes imaged emission lines to be tilted. In the original casing in [9], the slit could rotate freely, which we found difficult to control. Our casing fixes the slit in place when the casing is closed, which provides consistent slit orientation over imaging sessions. Furthermore, fixing the slit part of the S-tubes (2 in Figure 1), along with the collimator lens part (3 in Figure 1), allows adjustment of the focus tube during operation. A thumb hole was cut to the casing for the purpose.

Fixing holes in the bottom half of the casing fit to optical breadboards with 50 mm hole spacing for easy integration into optical laboratory equipment. For tripod usage, an adapter plate was devised (printable 3D model available at https://osf.io/srzgf).

The used video camera was Grasshopper3 model GS3-U3-91S6M-C (Flir) machine vision camera with ICX814 (Sony) monochromatic CCD sensor of 3376×2704 pixels. The 3D model of the casing must be modified if a different camera is being used. The camera heats up during operation, so a connection to air must be established to allow heat to dissipate. The orientation of the camera is forced to be parallel with the grating by the casing, so problems in orientating the camera with the slit image, as mentioned in [15], are avoided.

The used optical components are identical to those used in [9]. They are listed in Appendix A for completeness. From a monetary point of view, the used system costs less than 2000 euros, of which the machine vision camera costs roughly 1000 euros. Using cheaper camera and diffraction grating can bring the total price down to few hundred euros [8]. Recent development in 3D-printing imaging quality lenses [16] may lower the cost further in the future. A further benefit of using 3D-printed optics would be that, as it does not matter if normal or aberration corrected lens is printed, one could use better lenses in do-it-yourself imagers without additional cost and thus reduce the amount of aberrations.

The presented software artifact (available at https://github.com/silmae/desmiler) is capable of scanning hyperspectral image cubes, correcting spectral smile and tilt, and showing side-by-side comparisons of corrected and uncorrected data. For image cube comparison, there is a graphical user interface (GUI) with band selection and spectral angle mapping [17,18] views. Raw camera feed preview for tuning camera parameters and focus is also provided.

An effort was made to produce maintainable and modifiable program code, for example by using separate parameter script for dataset naming conventions and using an easily changeable interface for camera commands. We aimed for good coverage in documentation of the code. The software is written in Python programming language and it relies on publicly available libraries, most notably numpy [19], scipy [20], and xarray [21] for matrix calculations and data analysis, as well as open-source in-house camera controller library camazing (https://pypi.org/project/camazing).

### 2.1. Software Usage

The user interface (UI) of the software is implemented as an object of UI class used interactively through ipython [22] command shell. Instancing an object is simply done by running an ipython magic command %run ui.py, which will provide an UI object with name ui. Several synthetic examples (single frames and full image cubes) can also be generated and inspected using the UI object.

**Figure 3:**
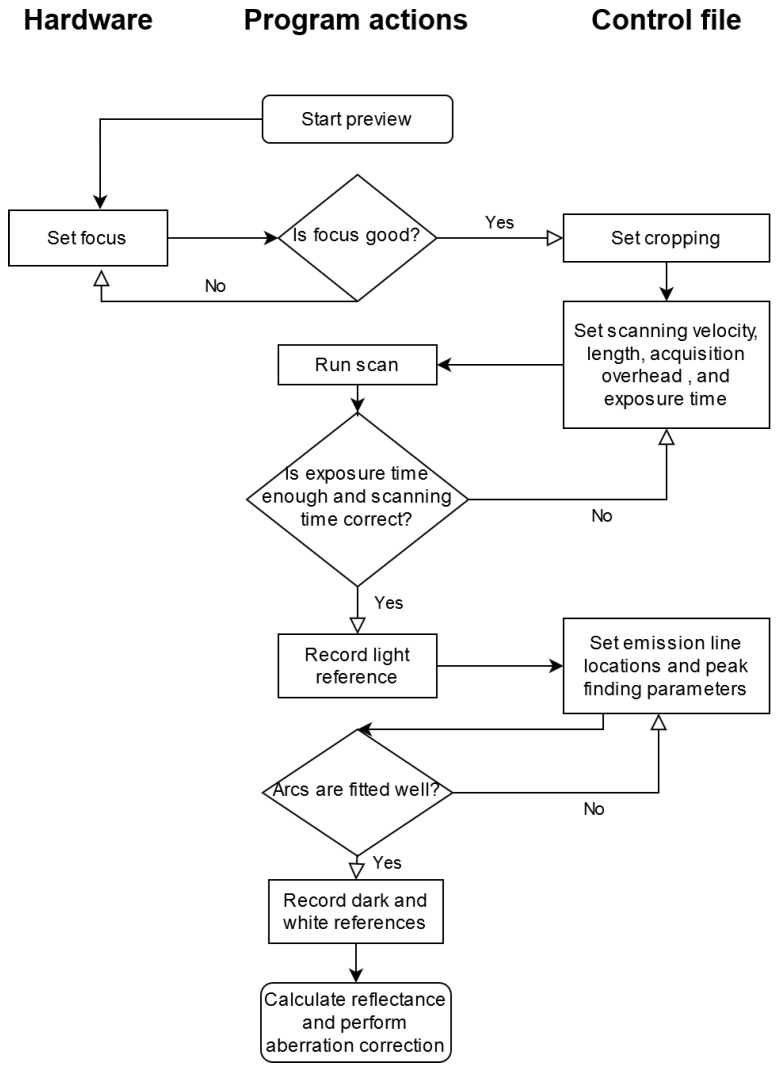
Flowchart of proposed imaging and aberration correction process. Hardware operations are located in the left-hand column, the center column holds software actions and the rightmost column represents settings written to the control file.

The flowchart in Figure 3 illustrates the operation of the software artifact. The only hardware operation is adjusting the focus with the help of the preview functionality. Operations used through the UI object are presented in the center column. There are several useful views helping the user with estimating the goodness of performed task (diamond shapes in the flowchart). Parameter tuning for scanning and aberration correction is done by writing desired values in a control file (right-hand column), which is reloaded from disk automatically when needed.

Preview is used to adjust the focus of the imager and cropping the raw sensor image to illuminated area. Scanning length and velocity can be given in arbitrary units, such as mm/s and mm, respectively. Acquisition overhead and exposure time affect how much time is allocated to acquire each frame and thus, the total frame count of the scan. The actual scanning time may differ if the imager cannot provide frames at requested rate, in which case the parameters need to be adjusted by increasing acquisition overhead or decreasing scanning velocity. Next, emission line location estimates and parameters for peak finding algorithm are given. After recording dark and white reference frames, aberration corrected reflectance image can be calculated.

### 2.2. Aberration Correction

Correcting the spectral smile is a well-researched subject (see, e.g., [11,12,13,14]), but we did not find any methods that could be applied without an optical laboratory. Our correction method is similar to that of Esmonde-White et al. [15] in that we search each row for pixels belonging to a certain emission line and transform the image so that curved lines become straight again. The major difference is that we do not need optical laboratory equipment for the correction. Our algorithm also takes the tilt effect into account and corrects both aberrations in a single pass. The main working principle is presented here, and an in-depth description is provided in Appendix B.

**Figure 4:**
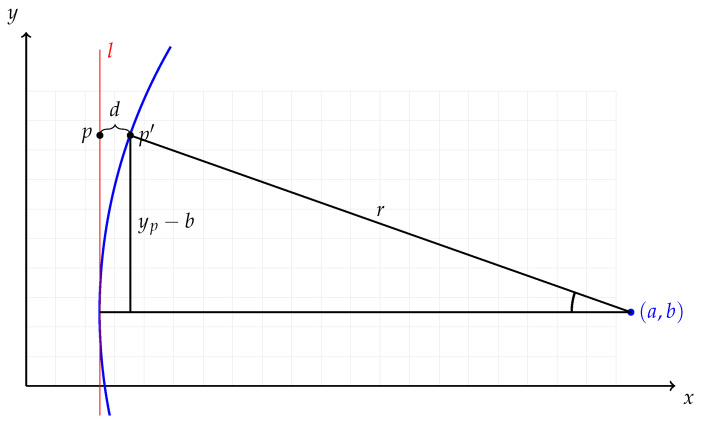
Emission line *l* is shown in red, and the fitted circular arc corresponding to it is shown in blue. The light gray grid represents the sensor of the camera. In reality, the radius of the circle would be several times larger than the width of the cropped frame acquired from the sensor.

Our smile correction method consists of three distinguishable steps: locating the emission lines of a frame, constructing a shift matrix, and applying the shift. In emission line location, we use a single frame with sharp emission lines and run a peak-finding algorithm for every row. The (x,y) positions of the peaks are ordered in sets, each belonging to a single emission line. A circular arc is then fitted to each set of pixel positions and its radius and center point are used to calculate an appropriate shift along each row needed to straighten the emission lines. These per-pixel shifts are stored in a shift matrix, which is used to alter the original frame to form a new, corrected frame. Figure 4 illustrates the matter. Some numerical considerations on the circular arcs and using curvature as a metric for straightness are presented in Appendix C.

Let us assume a photon that should have been registered in pixel $p=(x_{p},y_{p})$ was actually registered in pixel $p^{\prime }=\left(x_{p^{\prime }},y_{p}\right)$. Note that we assume the shift occurring only in spectral dimension, so the spatial *y*-coordinate remains constant.

Essentially, we want to know the distance between detected and true column of the photon $d=∥x_{p}-x_{p^{\prime }}∥$. Using the natural circle parameters, circle center (a,b) and radius *r*, which are given by the circle fitting, we get that the vertical distance between the circle center and pixel *p* is $∥y_{p}-b∥$. Using simple trigonometry, the distance from the circular arc to the line is

(1) $$d=r\left(1-\cos\left(\arcsin\frac{y_{p}-b}{r}\right)\right),$$

which is illustrated in Figure 4. It is easy to see that, if the circle center resides in the lower half of the frame, the distance *d* will be greater near the top of the grid than in the bottom. This asymmetry corrects the tilt effect as a side product.

We assume that the radius *r* of the circular arc is much greater than $∥y_{p}-b∥$, so the arcus sine is defined. This is a valid assumption, as, if it were not true, the frame would be distorted useless. With that assumption, we can say that, if sgn(a) is positive, the emission line curves to the right and the correct value can be obtained from pixel $\left(x_{p}+d,y_{p}\right)$, otherwise from pixel $\left(x_{p}-d,y_{p}\right)$.

Regardless of the position we move the emission line to, distances between emission lines are likely to change, which means that any wavelength calibrations must be done after the smile correction.

## 3. Experiments

To quantify the effectiveness of our method and show that the imager can be used without an optical laboratory, we conducted two experiments. In the first experiment, synthetic data were generated to show that our algorithm truly decreases the amount of spectral smile and the tilt. In the second experiment, a Macbeth color checker was scanned to show that hyperspectral image cubes can be acquired using just common office appliances.

### 3.1. Synthetic Frames

First, to construct an ideal, undistorted synthetic frame, a fluorescence light was imaged. The mean of several sensor rows was calculated to form a single smooth spectrogram, which acted as a base for the synthetic frame generation. This spectrum was then repeated on the synthetic sensor’s imaging area similar to real sensor. To generate non-identical, but similar spectra, each row was multiplied by a small random value drawn from a Gaussian distribution. In addition, random noise drawn from uniform distribution was added to each pixel value. The rest of the sensor rows, which would not get illuminated in the real sensor, were filled with random noise drawn from uniform distribution. The process is similar to the one used in [15]. A side-by-side comparison of a full synthetic frame and a real frame can be seen in Figure 2. For the rest of this section, we use only the illuminated (cropped) area of the sensor, as we would in the case of real frames.

**Figure 5:**
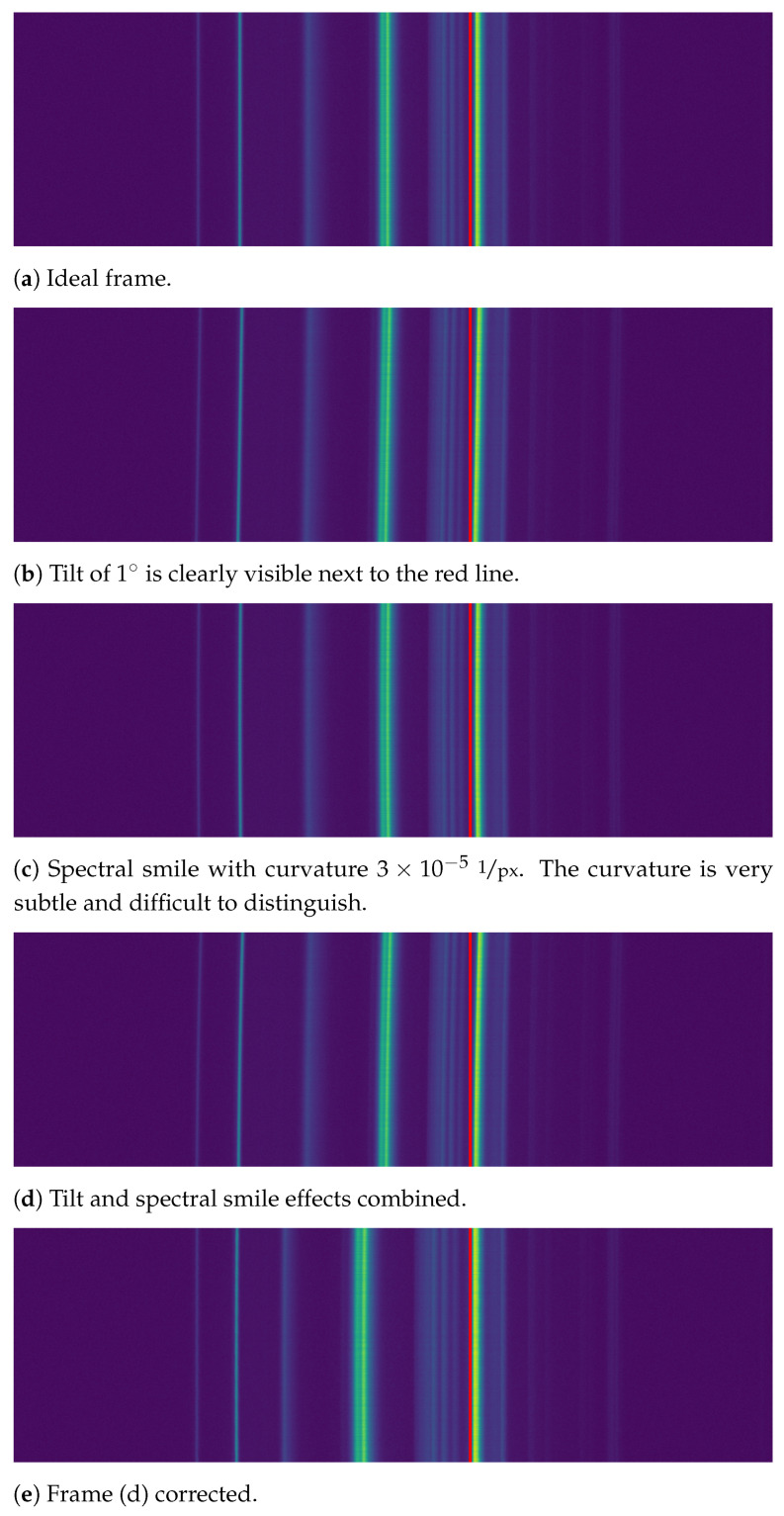
Examples of synthetic frames with distortions. As the effects are difficult to distinguish with the naked eye, the red line was added for aid. Frame (**a**) is an ideal frame that one would hope to get from a push-broom HSI. Frames (**b**,**c**) show the tilt and spectral smile aberrations, respectively. Frame (**d**) shows both aberrations combined, which represents the real data we get from the imager. Figure (**e**) shows Frame (**d**) after running the correction algorithm. The distortions are no longer distinguishable, but the emission line locations have shifted (see fourth line from the left).

The tilt and the spectral smile were generated using the correction algorithm in reverse, i.e., first generating a distortive shift matrix and interpolating each row with it. The tilt was set to $1^{\circ }$ and the curvature to $3\times 10^{-5}$ 1px, which are common values found in real frames acquired by our imager. Examples of the generated frames cropped to the imaging area with and without distortions can be seen in Figure 5.

For statistical analysis, 1000 frames were generated. The smile correction procedure was run for each frame, and the emission line search and curve fitting was used to determine the amount of tilt and spectral smile before and after the correction. Emission line search was successful for 937 frames. It is not likely that the actual correction had failed, but rather that the emission lines were not recognized well enough. However, the matter was not investigated further.

Tilt of the emission lines was estimated with linear least square error fit in (y,x) coordinate system; as the lines are nearly vertical, a line fit in (x,y) coordinates would have caused nearly infinite slope. Curvature of the emission lines was estimated using parabolic fit as in Equation (A5). Other possible curvature estimation methods are further considered in Appendix C. This experiment can be fully replicated using the program code available at https://github.com/silmae/desmiler.

### 3.2. Color Checker

**Figure 6:**
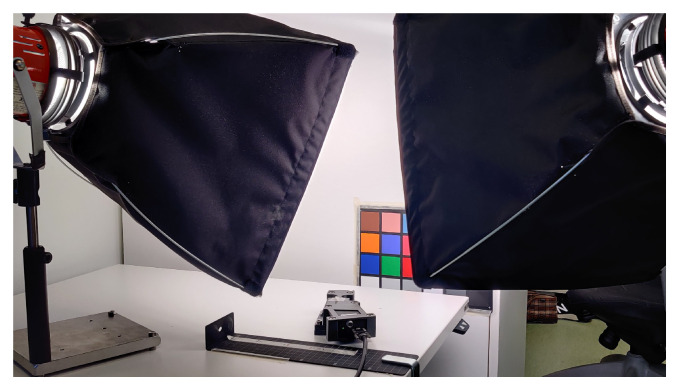
The used scanning setup: Two halogen lamps with a diffusor, color checker as an imaging target, and the hyperspectral imager, which was slid along the metallic ruler fixed to the desk.

In the color checker experiment, a Macbeth color checker (ColorChecker Classic, X-Rite) was selected as a test target. It was taped onto the side of an office locker along with a four-fold strip of common copy paper as a white reference target. The imager was placed on an office desk against a metallic ruler fastened to the desk with a clamp and some tape. Instead of using a motorized scanning platform, the imager was slid along the ruler by hand. Distance from the front lens to the target was approximately 30 cm, while the length of the scan was 14 cm. From that distance the imager could cover the squares and some of their black boundaries in across scan direction. Imaged area consisted of the red, green, and blue color tiles (4 cm × 4 cm each). 798 frames with 110 ms exposure time were recorded. Cropping was set to 2000 pixels in spectral dimension and 800 pixels in along slit direction. The scene was illuminated by two halogen lamps with a diffuser. The lamps were set above the imager on both sides, and they were immobile relative to the target. The experiment setup is shown in Figure 6.

## 4. Results

Comparing the ideal frame in Figure 5a with corrected frame in Figure 5e shows that our method corrects the tilt and spectral smile to imperceptible levels. The emission lines are shifted out of the original locations, as expected. Statistical results in Table 1 show that, on average, the tilt was reduced from $0.9^{\circ }$ to $0.005^{\circ }$, and spectral smile from $30\times 10^{-6}$ to $1\times 10^{-6}$. The uncorrected values in the table show the tilt is underestimated compared to the $1^{\circ }$ tilt used in the generation process.

One may notice that the error estimate values, the standard deviation of the mean, of the corrected frames are greater than the actual values, which implies that the correction reduces the aberrations to zero in terms of the used metrics. Numerical considerations of the curvature metric, presented in Appendix C, show that the curvature metric begins to fluctuate when values decrease below $10^{-6}$, so this behavior is expected.

The correction was based on four emission lines covering the range approximately from 400 to 600 nm. Emission Line 3 shows significantly greater error estimates than the other three lines. We suspect that the algorithm had troubles recognizing the line properly on all occasions, but the matter was not investigated further.

The correction shifted each emission line by 5 pixels on average on the used four emission lines. The shift of lines other than those used for the correction was not investigated, but some of the emission lines in Figure 5 seem to have shifted considerably more than this. The mere existence of the location shift implies that any wavelength calibration must be performed after the smile correction and not before.

**Figure 7:**
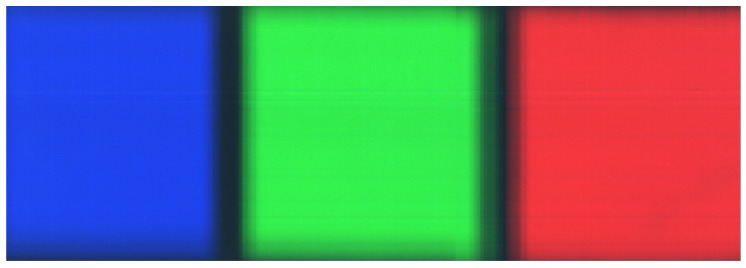
False color image constructed from the scan of the color checker. Scanning direction was from right to left. Blurry edges between the color tile boundaries are caused by uneven scanning speed of the manually driven scan. Vertical shift in the green tile is due to losing connection to the desk during the scan.

The false color reconstruction of the color checker scan in Figure 7 shows that the imager can provide decent scan quality without using optical laboratory. The black boundary between red and green tile is somewhat blurry, which is caused by the imager not being fully connected to the ruler. Vertical discontinuities near the right edge of the green tile are probably caused by the imager not being fully connected to the table during the scan. The faint horizontal stripes originate from unevenness of the slit.

## 5. Discussion

Our aberration correction method reduced both the spectral smile and tilt to zero within the accuracy of used metrics. The least squares fitting used as the core of both metrics sets the limit of the accuracy that can be achieved. The results should be further verified using well-established calibration methods, which is impossible with the equipment available in our laboratory.

Using the imager completely without laboratory equipment was shown to be possible, but using at least a motorized scanning platform is recommended for better quality scan results. In addition, as imaging in low lighting requires a long scanning time for reasonable signal-to-noise ratio, driving the scan manually can be quite straining. Using spectral calibration lamps with well-separated emission lines for light reference would lessen the manual labor required to find good emission line location estimates.

The software artifact is relatively easy to use, even if the user interface (UI) is a bit of a patchwork combining graphical elements, ipython console, and text file modification. However, using a very light UI makes it easy to add new features to the software and change the existing ones. The software was written in the viewpoint of correcting the spectral smile and lacks many features full imaging software should have; most notably, it lacks wavelength calibration, which is not essential for smile correction.

We made an effort to construct the software code in understandable and maintainable manner for ease of modification for different purposes. One such purpose might be to use this study as a student project for university level physics and information technology courses. For other than scientific audiences, we hope that easy-to-use software makes hyperspectral imaging more accessible and lessens the anxiety to try out hyperspectral imaging for various tasks that it can be useful in. The presented correction method removes the need for accessing an optical laboratory for spectral smile correction, and thus improves the image quality that can be expected from such low-cost imagers. We hope that this helps, in part, in adaptation of hyperspectral imaging as common tool rather than something only selected few with great resources can utilize.

For further research, the same circle fitting idea should be investigated in correcting the keystone aberration as well, as, even if it is a linear effect in itself, combined with the scaling effect that causes the red-end emission lines stretch in spatial dimension, the ends of the emission lines can be considered to form an arc. This is barely visible in Figure 2 as a sideways hourglass shape. We will continue to develop the software in the hope of eventually achieving full open-source imaging software beneficial to a greater group of users and adaptable to a large set of imagers.

## 6. Conclusions

In this study, we showed that spectral smile aberration of the push-broom hyperspectral imager presented by Sigernes et al. [9] can be corrected without using an optical laboratory. We also corrected an aberration, which we named tilt. The tilt is caused by the misalignment of the entrance slit and is specific for this particular imager design. We demonstrated that the imager can be used in acquisition of hyperspectral image cubes without access to an optical laboratory.

We provided simple open-source imaging software with the ability to correct spectral smile and the tilt and inspect the results. The software contains several built-in examples of spectral smile and tilt aberrations, and different kinds of examples can be generated by varying involved parameters. The program code is made publicly available through Github repository at https://github.com/silmae/desmiler under MIT license.

For those readers who wish to build their own imager, we provided required 3D models of the casing, in both print-ready and modifiable formats through https://osf.io/3uhkb. The repository also contains the dataset (hyperspectral image cube and reference frames for corrections) for reproducing the presented color checker experiment.

## Figures and Tables

**Table 1:** Mean values of tilt and curvature of 937 synthetic frames. Frames were generated with $1^{\circ }$ tilt and 3×10−5 1px curvature, which correlate well with mean values shown in original columns. Used error estimate is the standard deviation of the mean. Corrected values show that they are zero at the limit of the used metrics, as the error is greater than the values themselves. The four emission lines selected for the correction cover wavelengths approximately from 400 to 600 nm.

EL	Band	Tilt		Curvature	
		Original	Corrected	Original	Corrected
1	629	$\left(902\pm 13\right)\times 10^{-3}$	$\left(1\pm 7\right)\times 10^{-3}$	$\left(29.4\pm 2.5\right)\times 10^{-6}$	$\left(0.1\pm 1.2\right)\times 10^{-6}$
2	762	$\left(899\pm 19\right)\times 10^{-3}$	$\left(3\pm 79\right)\times 10^{-3}$	$\left(29.0\pm 3.5\right)\times 10^{-6}$	$\left(0.9\pm 10.3\right)\times 10^{-6}$
3	980	$\left(887\pm 35\right)\times 10^{-3}$	$\left(13\pm 107\right)\times 10^{-3}$	$\left(27.3\pm 6.7\right)\times 10^{-6}$	$\left(3.5\pm 23.3\right)\times 10^{-6}$
4	1517	$\left(902\pm 7\right)\times 10^{-3}$	$\left(1\pm 5\right)\times 10^{-3}$	$\left(29.7\pm 1.2\right)\times 10^{-6}$	$\left(0.1\pm 0.8\right)\times 10^{-6}$
Total mean		$\left(897\pm 14\right)\times 10^{-3}$	$\left(5\pm 14\right)\times 10^{-3}$	$\left(28.9\pm 2.5\right)\times 10^{-6}$	$\left(1.2\pm 2.6\right)\times 10^{-6}$

**Figure A1:**
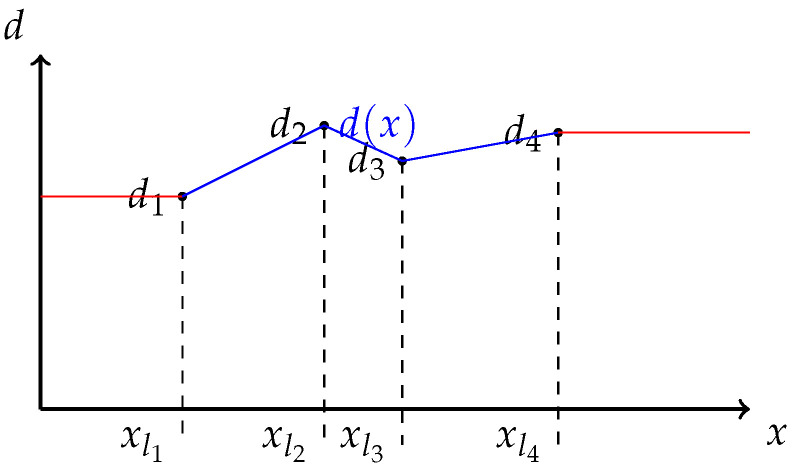
Distance function d(x) along a single row constructed using known distances for emission lines $l_{1},\ldots ,l_{4}$. Linearly interpolated region are shown in blue, extrapolated regions in red. In this case, extrapolation just assumes the closest known value.

**Figure A2:**
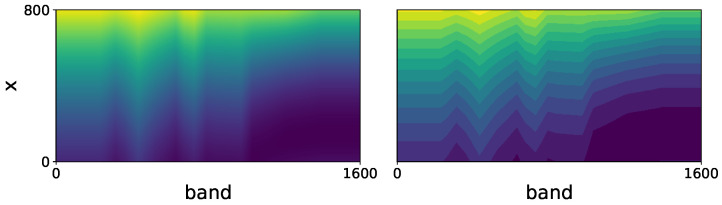
Frame-sized shift matrices where brighter color indicates greater shift distance: (**Left**) floating-point presentation; and (**Right**) values rounded to nearest integer. Rounding causes clearly visible discontinuations.

**Figure A3:**
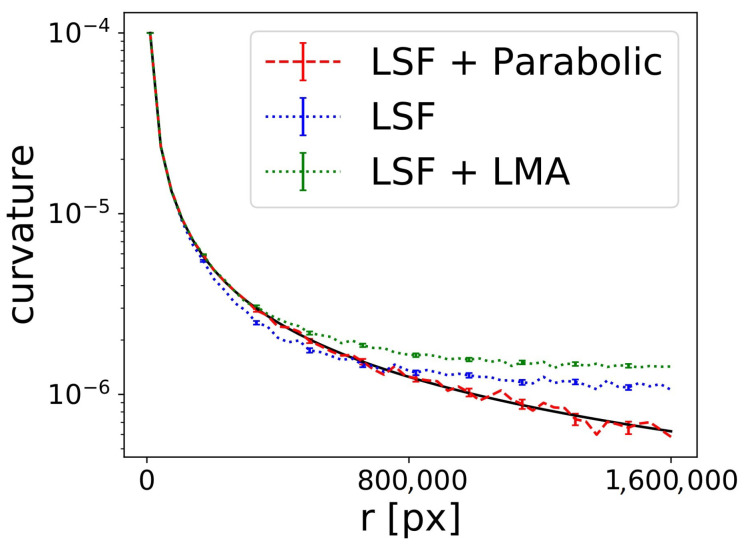
Synthetic emission line radius plotted against curvature acquired by different fitting methods. Parabolic fit is clearly the most stable and accurate of the three fitting methods when curvature is very low. The solid black line represents the ground truth 1r.

## Data Availability

The program code is available at https://github.com/silmae/desmiler under MIT license. The 3D models and dataset for the color checker experimet are available at https://osf.io/3uhkb under CC BY 4.0 license.

## Appendix A. Optical Components

Table A1 lists optical components needed to build used Sigernes design push-broom hyperspectral imager.

**Table A1:** Shopping list for the HSI. Adapted from [9]. Part numbers refer to the part numbers used on Edmund Optics website https://www.edmundoptics.com/.

Description	Part Number
Front lens f/4 Focal Length (FL) 16 mm	83–107
M12 lock nut for μ-video lenses	64–102
Precision air slit 25 μm × 3 mm	38–558
Field lens FL = 10 mm	63–519
3 × S-Mount thin lens mounts	63–943
S-mount focus tube	63–953
Collimator lens FL = 30 mm	63–523
600 grooves/mm transmission grating 25 × 25 mm^2^	49–580
Detector lens f/2.5 FL = 25 mm	56–776
C-mount to μ-video lens adapter	53–675

## Appendix B. Correction Algorithm Details

Our smile correction method is divided into three parts: locating the emission lines of a frame, constructing a shift matrix, and applying the shift. In emission line location, we use a single frame with sharp emission lines and run a peak-finding algorithm for every row. The (x,y) positions of the peaks are ordered in sets, each belonging to a single emission line. A circular arc is then fitted to each set of pixel positions and its radius and center point are used to calculate an appropriate shift along each row needed to straighten the emission lines. These per-pixel shifts are stored in a shift matrix, which is used to alter the original frame to form a new, corrected frame.

### Appendix B.1. Locating the Emission Lines

To construct a shift matrix, we first need a frame with sharp emission lines to base the correction on. A peak finding algorithm is used for every row in order to find the locations of the emission lines in the frame. We used signal.find_peaks() from SciPy library’s Signal package. We want to find only the most prominent emission lines so that there is a good chance to find the same peak from every row. To aid the peak finding algorithm, a band-pass filter is generated from user-defined emission line location estimates.

Once the row-wise peak positions are known, if the number of found peaks per row does not match the number of position estimates given by the user, the row is discarded. The mismatch occurs if the peak finding algorithm found zero or more than one peak within some filter window. They need to match because we have no other information on which peak should be associated with which emission line.

The number of points per line is not critical for the performance of curve fitting algorithms, which are discussed in Appendix C. Tests with synthetic data showed that the acceptance rate of 95% yields curvature error of approximately 0.3%, while 5% acceptance rate yields 1% error. The difference is very similar for all used fitting algorithms. Tests with real data displayed similar behavior when normal and averaged frames were compared: averaged frames have significantly better acceptance rate due to reduced noise.

After every row has the same number of peaks, they are arranged into emission lines (represented by SpectralLine class in the implementation) so that the first point of each row belongs to Emission Line 1, the second point to Emission Line 2, and so on. Now, each emission line is presented by a list of $\left(x_{i},y_{i}\right)$ points, where *i* is the index of an accepted row.

### Appendix B.2. Shift Matrix Construction

The next step is to construct the shift matrix *S* that stores the distances that each pixel has to be moved to be in their correct location in the spectral dimension. It has the same dimensions as the frame, and it is saved to disk to be used over several scanning sessions—until hardware changes are made. The shift matrix is the end-goal and the most important part of the smile correction. Each element $s_{i,j}\in S$ represents the distance needed to move along spectral dimension to find the correct value for each pixel $p_{i,j}$ in the original frame.

By selecting that the correct position of the line is at the furthest vertical distance from the circle center, we get a shift matrix where all values are either positive or negative. In the case of heavily tilted lines, the center point’s *y*-coordinate may not lie in frame’s area. It is then possible to have all of the emission lines to change location, as line *l* in Figure 4 will never touch the curve within frame’s area.

We establish a method for shift calculation in Equation (1) that assumes the smile to be consistent for every column of the frame. However, treating smile as a constant in the spectral dimension is not realistic as some lines are usually more heavily curved than others. Emission lines near the reference line would be well corrected, but lines further away possibly not. For example, if the correction were based on a heavily curved line, less curved lines elsewhere might get over-corrected, i.e., curved to the opposite direction.

If we can find more than one emission line from the frame, we can use the same method for each of them separately and interpolate over the whole area to form dissimilar columns to the shift matrix *S*. In other words, if we have two or more emission lines $\left\{l_{1},\ldots ,l_{n}\right\}$ into which we can fit corresponding circular arcs as before, we know the row-wise shift distance *d* at *n* points, which can be interpolated to form per-pixel distance function d(x) for each row separately. The distance function is illustrated in Figure A1.

The selection of extrapolation outside detected emission lines is of matter to small and high end of the spectrum. If a decent number of lines can be detected, the extrapolation may well lie in the area where sensor’s quantum efficiency drops and is not too critical for the resulting frame.

### Appendix B.3. Applying Shift

Once the shift matrix *S* has been constructed, we can apply it to the original frame acquired from the camera. We tested two methods of applying the shift: lookup table (LUT) and row-wise interpolation (INTR). In LUT method, the value of a pixel $p_{i,j}$ of a frame is simply replaced with the one at pixel $p_{i,j+s_{i,j}}$. INTR method interpolates each row of the frame based on corresponding row in *S*.

Distances in the shift matrix are expressed as floating-point numbers, but matrix indices need to be integers for the lookup table method. Casting or rounding the floating point into an integer is bound to cause discontinuities in the smile-corrected frame. Figure A2 visualizes how rounding affects the shift matrix. The left-hand image shows the shifts in the way they are stored, as floating-point numbers. In the right-hand side image, the distances are rounded to nearest integer, which causes the final smile-corrected frame to express the same kind of discontinuities and can be seen as jaggedness of emission lines. The effect is not visible when inspecting the image cube band-wise using the CubeInspector.

Differently from previous stages which need to be performed only once per system setup, the shift must be applied for every frame. In a full spectral cube, this may mean hundreds or thousands of frames, so the effect of computational cost becomes more prominent. LUT is a relatively simple operation using Numpy library’s re-indexing capability. INTR method requires much more complicated calculations and is considerably slower. We used Xarray Python library’s xarray.dataset.interp() method for interpolation.

It is also worth noting that, when LUT method is used, some pixels at the edge of the sensor are lost, as the shifted pixel coordinates will be outside of the frame area. This does not happen with interpolation, which alters the pixel values in-place. However, interpolation usually does generate NaNs (Not a Number) that have to be cleaned away as a post-process.

## Appendix C. Curvature as a Metric

Smile effect causes curving in detected emission lines on the sensor, which can be clearly seen from the real frame shown in Figure 2. If we approximate a curved emission line with a circular arc, we can use reciprocal of the radius as a metric for curvature. As the emission lines are formed by a set of points, i.e., locations of spectral peaks along each sensor row, we can fit a function to that point set to gain function parameters best describing the shape.

To estimate the performance of our method, we need to be able to measure the curvature after smile correction. Fitting a circular arc to a nearly straight line accurately is not a completely trivial task [23]. We compare the accuracy and robustness of three curvature estimates produced by different fitting methods.

### Appendix C.1. Least Squares Circle Fitting

One of the simplest circle fitting methods is the least squares fitting (LSF). If given *n* points $\left(x_{i},y_{i}\right)$ where i∈[1,n], LSF becomes the minimization problem of the sum of squared distances from the arc. A circle can be defined by

$${\left(x-a\right)}^{2}+{\left(y-b\right)}^{2}=r{\;}^{2},$$

where (a,b) is its center point and *r* is its radius. The unrestricted non-linear minimization problem for fitting is then

(A1) $$\min_{a,b,r}\sum d_{i}^{2},$$

where $d_{i}$ is the Euclidean distance of each point to the circle

(A2) $$d_{i}=\sqrt{{\left(x_{i}-a\right)}^{2}+{\left(y_{i}-b\right)}^{2}}-r.$$

As can be seen from this parameterization, when the arc has low curvature, i.e., large radius, the parameters become arbitrarily large, which leads to catastrophic loss of accuracy in Equation (A2) where two large and nearly equal values are subtracted from each other.

### Appendix C.2. LMA Fitting

Chernov and Lesort [23] suggested changing the parameterization of the optimization problem for better stability. They suggested using Pratt’s circle parameterization [24], which defines a circle by

(A3) $$A\left(x^{2}+y^{2}\right)+Bx+Cy+D=0,$$

where the first term describes a circle and the remaining three terms form an equation of a line. Conversion formulas to natural circle parameters *a*, *b*, and *r* are

(A4) $$A=\pm \frac{1}{2r},\;B=-2Aa,\;C=-2Ab,\;D=\frac{B^{2}+C^{2}-1}{4A}.$$

For best optimization results, Chernov and Lesort [23] suggested using unrestricted Levenberg–Maquardt corrected Gauss–Newton method with three-dimensional parameter space (A,D,θ), where θ is defined by

$$B=\sqrt{1+4AD}\cos\theta ,\;C=\sqrt{1+4AD}\sin\theta .$$

The distance to be minimized in Equation (A1) is

$$d_{i}=\frac{2P_{i}}{1+\sqrt{1+4AP_{i}}},$$

where

$$P_{i}=A\left(x_{i}^{2}+y_{i}^{2}\right)+\sqrt{1+4AD}\;\left(x_{i}\cos\theta +y_{i}\sin\theta \right)+D.$$

This parameterization ensures that, even when approaching a line, none of the parameters explode. A line would just mean that A=0.

For partial differentials needed to form the Jacobian matrix for the optimizer, refer to the work of Chernov and Lesort [23].

### Appendix C.3. Parabolic Least Squares Fitting

For a sideways opening parabola

(A5) $$x=ay^{2}+by+c,$$

the radius of curvature at its vertex has the same length as its semi-latus rectum r=12a so the curvature is

$$\frac{1}{r}=2a.$$

This formulation does not allow arbitrary opening direction, but, assuming the tilt is only a few degrees, it does not pose a problem. Parameter *b* allows the vertex of the parabola to move along *y*-axis, thus it gives us the curvature at the point of greatest curvature. As factor *a* approaches zero, the equation becomes that of a line x=by+c, which is the tangent of the parabola at the intersection with *x*-axis.

### Appendix C.4. Optimization and Accuracy

For testing the performance of the three fitting methods and the curvature values they produce, synthetic data were generated. Generated data represent curved emission lines in *x*, *y* coordinates, where the lines are aligned along the *y*-axis. The *x*-coordinate was kept constant and small, which forces circle fitting parameters *a* and *r* to grow together when radius is increased. One hundred real frames were analyzed beforehand and extracted averages were used for synthetic data generation parameters.

A single synthetic emission line was generated by first generating 800 data points along a perfect circular arc. Their *x*-coordinate was then disturbed by normally distributed noise with variance $\sigma^{2}=1.5$. Then, 95% of the points were randomly discarded using uniform distribution, which represents usual discard rate when real singular frames are acquired from the imager.

Least squares fitting was performed with scipy.optimize.leastsq() using the barycenter of the data as a starting guess for the center point.

LMA was optimized with scipy.optimize.least_squares(), which includes an implementation of Levenberg–Maquardt corrected Gauss–Newton method for unconstrained optimization problems. The result of the previously run LSF was used as a starting guess for *a*, *b*, and *r*, which were converted to *A*, *D*, and θ using formulas in Equation (A4).

Parabolic least squares fit was optimized with scipy.optimize.curve_fit() method, with parameters *a*, *b*, and *c* as in Equation (A5). A starting guess for parameter *a* was obtained from previously run least squares circle fit a=12r. The other starting parameters were set so that *c* is the mean of *x*-coordinates and b=0.001, which is essentially just an arbitrary small number.

Figure A3 shows the simulation results for emission lines generated with increasing radius. Fifty different radii were used for all three curve fitting methods. The lines represent the mean of 1000 runs and error bars as standard error of the mean are plotted for every fifth radius.

The LSF method begins to deviate from the ground truth very early. It gives consistently too small values when r≤700,000 and too great values after that. The LMA method is fairly accurate when r≤500,000 and consistently overestimates the curvature thereafter. Parabolic fit is the most consistent with the ground truth. It begins to oscillate around the ground truth at approximately same radius where the LMA method starts to deviate, but it does not show any systematic deviation as the other two.

The smallest curvatures in our uncorrected experiment data were $10^{-5}$ in magnitude; thus, in terms of accuracy, the smile correction can be based on any of the three fitting methods.
